# Blackthorn—A Valuable Source of Phenolic Antioxidants with Potential Health Benefits

**DOI:** 10.3390/molecules28083456

**Published:** 2023-04-14

**Authors:** Oana-Raluca Negrean, Anca Corina Farcas, Oana Lelia Pop, Sonia Ancuta Socaci

**Affiliations:** 1 Department of Food Science, University of Agricultural Science and Veterinary Medicine of Cluj-Napoca, 400372 Cluj-Napoca, Romania; 2 Molecular Nutrition and Proteomics Lab, CDS3, Life Science Institute, University of Agricultural Science and Veterinary Medicine of Cluj-Napoca, 400372 Cluj-Napoca, Romania; 3 Life Science Institute, University of Agricultural Science and Veterinary Medicine of Cluj-Napoca, 400372 Cluj-Napoca, Romania

**Keywords:** antioxidant, antibacterial properties, bioactivities, blackthorn, *Prunus spinosa* L.

## Abstract

*Prunus spinosa* L. fruit, commonly known as blackthorn, is a rich source of bioactive compounds, including flavonoids, anthocyanins, phenolic acids, vitamins, minerals, and organic acids, which exhibit significant antioxidant and antibacterial properties. Notably, flavonoids such as catechin, epicatechin, and rutin have been reported to have protective effects against diabetes, while other flavonoids, including myricetin, quercetin, and kaempferol, exhibit antihypertensive activity. Solvent extraction methods are widely used for the extraction of phenolic compounds from plant sources, owing to their simplicity, efficacy, and broad applicability. Furthermore, modern extraction techniques, such as microwave-assisted extraction (MAE) and ultrasound-assisted extraction (UAE), have been employed to extract polyphenols from *Prunus spinosa* L. fruits. This review aims to provide a comprehensive analysis of the biologically active compounds found in blackthorn fruits, emphasizing their direct physiological effects on the human body. Additionally, the manuscript highlights the potential applications of blackthorn fruits in various industries, including the food, cosmetics, pharmaceutical, and functional product sectors.

## 1. Introduction

Widely cultivated in New Zealand, Tasmania, and eastern North America, *Prunus spinosa* L., known as blackthorn or sloe berry, is a small to medium-sized thorny tree native to Europe, Western Asia, and Northern Africa [1]. The tiny, globose blackthorn fruits have an astringent flavor and a deep purple color. They are usually used to make jams, drinks, and supplements and can be preserved and used to make herbal teas [1,2,3].

Blackthorn contains a variety of bioactive polyphenolic compounds, including phenolic acids (neo-chlorogenic and caffeic derivatives), flavonoids (rutin, catechins, procyanidins, quercetin, kaempferol), and anthocyanins [4,5,6,7,8,9,10], nor-isoprenoid glycosides, and A-type proanthocyanidins [11] along as well as ascorbic acid, carbohydrates, macro and microminerals [2,12,13].

Flavonoids, particularly anthocyanins, are known for their potential health benefits due to their antioxidant properties. Studies have suggested that consuming foods with high antioxidant levels, such as flavonoids, can lower the risk of developing various diseases. Therefore, including anthocyanin-rich foods in one’s diet can have a significantly positive impact on human health [14,15].

According to ethnopharmacological investigations, blackthorn fruit has been used to treat external inflammatory conditions in the mouth and throat, diabetes, pneumonia, and diarrhea [16,17,18]. For many years, humans have used the leaves and fruits of blackthorn in traditional medicine as a laxative and the flowers as a natural vermicidal treatment [19]. It was also mentioned that due to its various bioactive components, a different part of the blackthorn plant has historically been used to stimulate and regulate the menstrual flow and function and treat leucorrhoea, but also for their analgesic, antispasmodic, anti-edema, and antidysenteric properties [20].

Furthermore, a series of recent researches have shown that blackthorn aqueous or alcoholic extracts have significant antioxidant, antibacterial (against both Gram-positive and Gram-negative bacteria), anti-inflammatory activities, and more than that, they have cancer cell growth-inhibiting effects [8,19,21,22,23,24].

**Figure 1:**
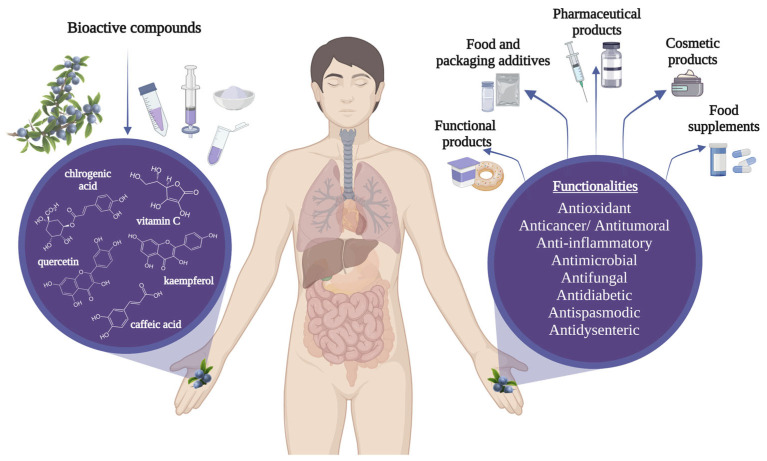
Blackthorn applications and potential health properties.

Figure 1 illustrates the main applications of blackthorn across diverse domains, alongside their potential health-promoting properties, which will be discussed in detail in the following sections.

Blackthorn extracts may be used in various areas, including the food industry, as natural colorants and as preservatives because of the polyphenols’ antioxidant and antibacterial properties [8,25,26,27]. For example, blackthorn fruits added to ice cream samples improved qualities such as color, gumming structure, appearance, and general acceptability [28,29]. Blackthorn fruits are recognized as an excellent source of vitamin C, with numerous studies reporting a value of around 25 mg 100 g^−1^ fw [3,30,31]. Moreover, the plant possesses significant quantities of macro and microelements such as calcium (19.859–34.234 µg/g dw), potassium (1202.822–18,706.98 µg/g dw), iron (3.399–16.18 µg/g dw), sodium (530.11 µg/g dw), zinc (0.350–1.803 µg/g dw), manganese (1.868–4.58 µg/g dw), and magnesium (8.574–11.827 µg/g dw) [2,12]. Băbălău-Fuss et al., 2021 determined that blackthorn oil has oleic acid as a majority fatty acid. Additionally, it was shown in this study that blackthorn oil presents a total concentration of mono and polyunsaturated fatty acids of 80.74% [32]. Because they have a high content of beta-sitosterol, vanillin, and gamma-tocopherols, cold press oils may have various applicability in food, health, and pharmaceutical industries [33,34]. The blackthorn extracts are also suitable for cosmetic use because of their photoprotective effects [35]. At wavelengths between 200 and 400 nm, phenolic compounds present a significant role in UV ray absorption [36].

Another application of blackthorn fruit extracts is in the food industry. Mandic et al. (2018) developed a study on incorporating extract of blackthorn fruits in the natural casing of Kranjska sausages. This study showed that the blackthorn extracts, both aqueous and ethanol, succeeded in reducing the number of lactic acid bacteria from the surface of vacuum-packed sausages that were stored at 4 °C for 60 days [37].

In this paper, our objective is to conduct a comprehensive analysis of the biologically active compounds present in blackthorn fruits, highlighting their primary physiological effects on the human body. Additionally, we aim to emphasize the beneficial properties of blackthorn fruits in their potential application across various industries, including the food, cosmetic, and pharmaceutical sectors, as well as in the development of functional products.

### The Methodological Approach

The methodology employed in this bibliographic research is a narrative review, which entails conducting a thorough search of pertinent literature related to the research topic, followed by an evaluative assessment of the identified studies. The aim is to synthesize the existing evidence to highlight the potential applications of blackthorn. The review draws information from academic databases, specifically PubMed, Scopus, and Web of Science, while excluding grey literature sources such as conference proceedings, institutional repositories, and government reports. The studies were selected based on their relevance to the research topic, the quality of their design and methodology, and the availability of data. Studies that did not meet these criteria, such as those not relevant to the research topic, lacking a control group, or not written in English, were excluded. The search strategy utilized a combination of keywords such as “blackthorn”, “*Prunus spinosa*”, “antioxidants”, “flavonoids”, “polyphenols”, “health benefits”, “anti-inflammatory”, “anti-cancer”, and “immune system”, along with Boolean operators.

## 2. Bioactive Compounds

The blackthorn fruit is a rich source of components with antioxidant and antibacterial characteristics, including flavonoids, anthocyanins, phenolic acids, vitamins, minerals, and organic acids [29,38]. Among these components, polyphenols are particularly noteworthy for their strong contribution to the antioxidant capacity of fruits and vegetables compared to other antioxidant compounds, such as vitamins C and E or carotenoids [39,40,41]. Phenolic phytochemicals are also of major impact on defense responses, including anti-aging, anti-inflammatory, antioxidant, and antiproliferative effects [42]. Therefore, the abundance of polyphenols and other antioxidants in blackthorn fruit highlights its potential as a valuable resource for preventing oxidative stress and related health issues.

**Figure 2:**
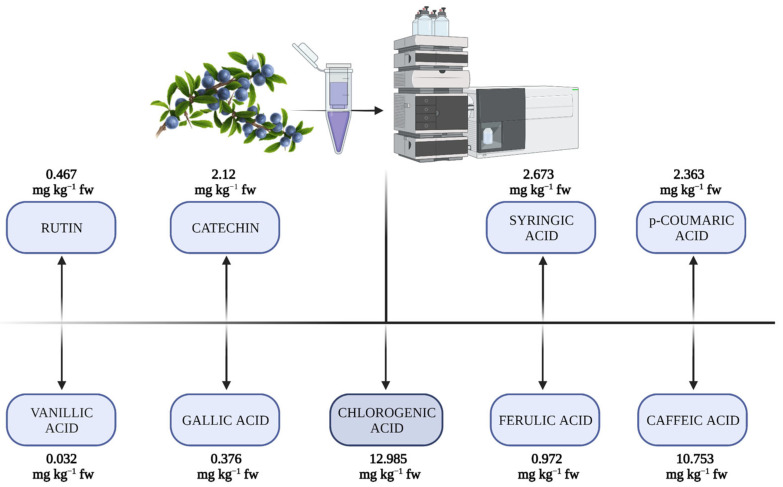
The main phenolic compounds from blackthorn fruits (adapted from [31]).

The most common flavonoids in these fruits are quercetin and its glycosides, such as rutin, which are found in higher concentrations in the peel [43]. In previous studies, substances such as quercetin, cyanidin, caffeic acids, catechin, rutin, epicatechin, kaempferol, gallic acid, chlorogenic acid, syringic acid, vanillic acid, ferulic acid, p-coumaric acid were found in blackthorn [2,10,19,31,44]. Gallic acid has powerful anti-oxidative, anti-inflammatory, antibacterial, antiviral, anti-melanogenic, antimutagenic, and anticancer properties [45,46,47]. The most representative phenolic compounds found in blackthorn fruits and their quantities are displayed in Figure 2.

It is well known that quercetin glucoside bioavailability is significantly higher than quercetin rutinoside bioavailability, indicating that the small intestine actively absorbs the glucosides [44]. Due to its complete structural functionality and high radical scavenging capacity, quercetin functions as a potent antioxidant [48,49]. Caffeoylquinic acid and quercetin were the dominant compounds in Marcetic et al.’s (2022) study of phenols in blackthorn fruit extracts [50]. The primary phenolic acids found in blackthorn fruits are neochlorogenic and chlorogenic acid (3-caffeoylquinic acid) [43,51,52,53,54,55]. The only flavonoid found by Najgebauer-Lejko et al. (2021) in blackthorn puree was myricetin. They also remarked that chlorogenic (144.98 mg/100 g DW) and caffeic acids (100.39 mg/100 g DW) were the most abundant compounds among phenolic acids [56].

Previously, researchers from Northeastern Portugal discovered that among three samples of wild fruits, strawberry tree, blackthorn, and wild rose, blackthorn fruits were the only ones that contained phenolic acids from the hydroxycinnamic acid derivate subgroup. Blackthorn fruits had the highest levels of phenolic acids (29.78 mg/100 g), flavone/ols (57.48 mg/100 g), and anthocyanins (100.40 lg/100 g), despite the absence of flavan-3-ols compounds [5].

Mechchate et al. (2021) reported that catechin, epicatechin, and rutin were found to be potent antihypertensive flavonoid agents [57]. In addition, rutin, myricetin, quercetin, and kaempferol protect against diabetes [58]. In isolated arteries, quercetin and similar flavonoids have a vasodilation action that is both endothelium-dependent and independent [59,60]. Blackthorn fruits have an astringent flavor because of the tannins that are present there. Like many other polyphenols, tannins have various biological effects, including anti-inflammatory, anti-carcinogenic, anti-microbial, and protective of the cardiovascular system [61,62,63].

The colorful fruits are a rich source of antioxidant compounds such as phenolic pigments and anthocyanins, which have numerous health benefits [64,65]. The high anthocyanin concentration gives blackthorn (*Prunus spinosa* L.) fruit its dark blue hue. Anthocyanins are a sizable subclass of flavonoids that produce vibrant shades ranging from scarlet to blue or red to orange [66]. Cyanidin is the main anthocyanin found in stone fruits belonging to the Prunus genus. Anthocyanins’ advantages for cardiovascular health have been connected to their capacity to fend off oxidative stress [67]. Katanic Stankovic et al. (2022) found a substantial amount of anthocyanins (461.27 mg cyanidin-3-glucoside eq/kg fresh fruit) in an ethyl acetate extract of Serbian blackthorn fruits, equivalent to aronia. The HPLC analysis was used to identify phenolic acids and anthocyanins from investigated extracts [68]. Sabatini et al. (2020) took into account the hypothesis that the great efficacy in the anti-microbial effect of blackthorn fruits may be due to anthocyanins or the combined action of bioactive compounds [69]. The anthocyanin components cyanidin-3-*O*-glucoside, cyanidin-3-*O*-rutinoside, and peonidin-3-*O*-glucoside were found in the aqueous extract by Veličković et al. (2014), while phenolic acids (neochlorogenic and caffeic acid) and flavonoids were present in the ethanol and ethanol-aqueous extracts (myricetin and quercetin) [10]. Mineral or organic acid-containing solvents are commonly used to extract anthocyanins from plant organs [70]. Anthocyanins are likely substantial contributors to the antioxidant activities of blackthorn leaves, as evidenced by correlations between quantities of phenols, flavonoids, and anthocyanins and values obtained for antioxidant activity established in a previous study [71].

According to Wang et al. (2022), anthocyanins could prevent the growth of dangerous bacteria by disrupting respiratory metabolism and inhibiting gene expression. Furthermore, by stimulating vital enzyme activities, the presence of anthocyanins may promote the growth of probiotics [72].

Pinacho et al. (2015) conducted a study to evaluate the phenolic compounds of blackthorn and their antioxidant capacity, specifically examining the impact of in vitro digestion. The researchers found that branches of the plant exhibited a higher antioxidant capacity compared to other plant components (leaves and fruits). To investigate the effect of digestion, an ethanolic extract of blackthorn branches was subjected to in vitro oral, gastric, and intestinal digestion. Results indicated that no significant changes occurred during oral or gastric digestion, suggesting the stability of the phenolic compounds under these conditions. However, intestinal digestion resulted in extensive degradation of the chemicals, likely due to the alkaline conditions in the small intestine. The researchers observed the formation of various structural forms with significant antioxidant activity, some of which were previously unknown or unrecognized. Based on their findings, the researchers concluded that the blackthorn extracts could potentially serve as a valuable resource for preventing oxidative stress-related illnesses [73].

## 3. Extraction Methods

The chemical nature of the phenolic compounds extracted from plant parts, the sample matrix structure, the time of extraction, the storage condition, and the presence of any interfering substances are influencing more or less the extraction efficiency. Plant phenolic extracts are a mixture of different classes of phenols that are selectively soluble in different solvents [74]. Because of their ease of use, efficiency, and broad applicability, solvent extractions are the most used procedures to extract phenolic compounds from plant sources and free them from the vacuolar structures where they are found [75]. The type of solvent used and how polar it is, the temperature and length of the extraction process, as well as the chemical and physical characteristics of the materials being extracted, all affect chemical extraction [75]. For phenolic extraction, typical solvents include water, acetone, methanol, ethanol, *N*,*N*-dimethylformamide (DMF), or combinations of these substances in water. Their extraction efficiency has been studied due to polarity differences [76,77]. Methanol, for example, has some disadvantages, such as potentially hazardous effects on human health. Its residues may remain in the final product, requiring additional time-consuming purification steps that influence the final cost of the process. On the other hand, if for extraction are used pure organic solvents such as benzoic or cinnamic acid, extraction may not be completed [70].

**Figure 3:**
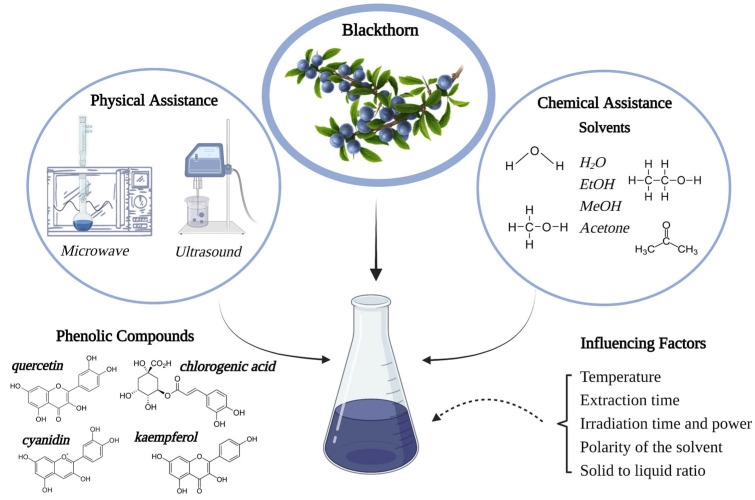
The main extraction techniques and factors affecting recovery yield of phenolic compounds from blackthorn.

Additionally, modern extraction methods have recently been used to facilitate the isolation of polyphenolic compounds (accelerated solvent extraction, microwave-assisted extraction, and supercritical fluid extraction) [78]. The main techniques currently used for the extraction of phenolic compounds from blackthorn, as well as the factors that influence the recovery yield, are summarized in Figure 3.

High-performance liquid chromatography (HPLC) and ultra-high-performance liquid chromatography (UHPLC) are the most frequently used methods for polyphenol separation. Liquid chromatography is often combined with mass spectrometry (LC-MS) for more selective and reliable compound identification, as reported by Rajbhar et al. (2015). Also, thin-layer chromatography (TLC), capillary electrophoresis (CE), and gas chromatography (GC) can be applied for separating individual polyphenolics from fruit samples [74,79].

Besides the importance of quantification of individual polyphenols, total phenolic content, and methods for determining antioxidant activity are important. The Folin-Ciocalteu assay is used to determine total phenolic content (TPC), with the calibration curve typically constructed using gallic acid as a standard [65,79].

Quantifying polyphenolic compounds is a challenging and complex process that requires careful consideration of various factors. In order to better understand the extraction of phenols from blackthorn, several studies have been conducted to evaluate the working parameters and extraction yield. Therefore, some studies related to the extraction of phenols from blackthorn, as well as the working parameters and the extraction yield are summarized in Table 1.

Modern extraction methods such as microwave-assisted extraction (MAE) and ultrasound-assisted extraction (UAE) are often used to increase the extraction yield of polyphenols from plants. UAE can improve mass transfer, cell disruption, penetration, and capillary effects [74,80]. In addition, MAE’s benefits require little or no solvent, reduce extraction time, and are environmentally friendly [81,82].

**Table 1:** The extraction of phenols from different blackthorn parts and the applied protocols.

Plant Part	Conditions and Effectiveness of Extraction	Antioxidant Content			References
		TPC	TFC	TAC	
SOLVENT EXTRACTION					
Leaves	Distilled water Yield: 13.65%	142.40 ± 3.82 mg GAE/g dw	36.28 ± 0.41 mg QE/g dw	-	[71]
	Ethanol 96% Yield: 9.14%	116.63 ± 1.62 mg GAE/g dw	45.52 ± 0.9 mg QE/g dw	-	
	Acetone Yield: 4.36%	181.19 ± 1.70 mg GAE/g dw	80.10 ± 0.00 mg QE/g dw	-	
Fruit	Distilled water Yield: 18.45%	23.19 ± 2.52 mg GAE/g dw	2.96 ± 0.22 mg QE/g dw	14.00 µg/g dw	[83]
	Ethanol 96% Yield: 11.09%	19.98 ± 1.28 mg GAE/g dw	3.07 ± 0.27 mg QE/g dw	9.00 µg/g dw	
	Acetone Yield: 9.4%	26.78 ± 4.44 mg GAE/g dw	2.89 ± 0.36 mg QE/g dw	23.00 µg/g dw	
	Methanol/Water (50:50, pH 2) Acetone/Water (70:30)	37.97 ± 0.30 mg GAE/g fw	2.26 ± 0.15 mg RUE/g fw	25.85 ± 1.51 mg pelargonidin 3-glucoside/g fw	[30]
Flower	Methanol/Water (7:3, *v/v*)	206.07 ± 10.86 mg GAE/g dw	125.12 ± 0.55 mg/g dw	45.13 ± 2.38 mg CYE/g dw	[84]
	Diethyl ether Yield: 1.23 g dw	464.57 ± 20.57 mg GAE/g dw	490.63 ± 8.16 mg/g dw	49.5 ± 2.23 mg CYE/g dw	
	Ethyl acetate Yield: 4.00 g dw	584.07 ± 12.98 mg GAE/g dw	325.53 ± 4.23 mg/g dw	109.43 ± 3.71 mg CYE/g dw	
	n-butanol Yield: 4.86 g dw	296.57 ± 3.28 mg GAE/g dw	241.27 ± 4.74 mg/g dw	46.6 ± 1.14 mg CYE/g dw	
	Water residue Yield: 13.08	64.6 ± 1.93 mg GAE/g dw	1.88 ± 0.04 mg/g dw	12.43 ± 0.25 mg CYE/g dw	
MICROWAVE-ASSISTED EXTRACTION					
Flowers	Ethanol 1 min 60 °C	54.45 ± 0.12 mg GAE/g dw Borije	1.547 ± 0.001 mg QE/g dw Vareš	0.339 ± 0.063 mg CGE/g dw Trnovo	[2]
Leaves	Ethanol 1 min 60 °C	17.78 ± 0.10 mg GAE/g dw Borije	0.479 ± 0.001 mg QE/g dw Vareš	1.353 ± 0.060 mg CGE/g dw Trnovo	
Fruits	Ethanol 1 min 60 °C	6.87 ± 0.01 mg GAE/g dw Borije	0.149 ± 0.001 mg QE/g dw Vareš	0.746 ± 0.092 mg CGE/g dw Trnovo	
ULTRASOUND-ASSISTED EXTRACTION					
Fruit	Ethanol 75% Ultrasound bath: 240 W, 35 kHz 25 °C for 30 min	25.9 ± 0.2 mg GAE/g fw	5.09 ± 0.12 mg RUE/g fw	0.16 ± 0.001 mg Mv-3- glc/g fw	[68]
	Ethanol 40% Ultrasound bath: 95 W, 35 kHz 67 °C for 10 min	1.02 mg GAE/g dw	-	-	[35]
	Ethanol Ultrasound bath: 20 min	4.116 ± 0.003 mg GAE/g dw Borije	0.064 ± 0.001 mg QE/g dw Vareš	1.258 ± 0.029 mg CGE/g dw Trnovo	[2]
Flowers	Ethanol Ultrasound bath: 20 min	24.41 ± 0.03 mg GAE/g dw Borije	0.677 ± 0.001 mg QE/g dw Vareš	0.718 ± 0.058 mg CGE/g dw Trnovo	
Leaves	Ethanol Ultrasound bath: 20 min	8.31 ± 0.03 mg GAE/g dw Borije	0.282 ± 0.001 mg QE/g dw Vareš	1.364 ± 0.03 mg CGE/g dw Trnovo	

TPC—Total Phenolic Content; TFC—Total Flavonoid Content; TAC—Total Anthocyanin Content;. GAE—gallic acid equivalent; QE—quercetin equivalent; CYE—cyanidine chloride equivalents; CGE—cyanidin-3-glucoside equivalents; RUE—rutin equivalent; Mv-3-glc—malvidin-3-glucoside; dw—dry weight; fw—fresh weight.

The studies detailed in Table 1 highlight that ethanol is the most commonly used solvent to extract chemical compounds from different parts of blackthorn. However, it has been demonstrated that phenolic compounds are extracted more effectively in acetone than in ethanol. For example, the fruit TPC of the acetone extract was 26.78 ± 4.44 mg GAE/g, while for the ethanol extract was 19.98 ± 1.28 mg GAE/g [83]. On the other hand, despite the higher TPC in the fruit acetone extract, the ethanolic extract showed higher in vitro antioxidant and antibacterial properties, as well as a potential antidiabetic action. Additionally, the total phenolic content determined in the ethanolic extract using conventional methods showed a higher content (19.98 ± 1.28 mg GAE/g [83]) than microwave-assisted extraction (6.87 ± 0.01 mg GAE/g [2]).

## 4. The Potential Effects of Bioactive Compounds

### 4.1. Antioxidant Activity

Antioxidants are commonly integrated into preservation procedures to enhance stability by mitigating the deleterious impact of reactive oxygen species on products as well as in the human body [85,86]. Anthocyanins, which are found in blackthorn, comprise one of the main classes of water-soluble flavonoids that contribute to flavonoids’ antioxidant activities [87]. Wang and Lin (2000) discovered a strong relationship between antioxidant capacity (oxygen radical absorbance capacity, ORAC), anthocyanins (pH differential method), and total phenols (spectrophotometric method, using Folin-Ciocalteu reagent) [88]. Fraternale et al. (2009) researched the antioxidant activity of blackthorn fruit juice. They compared the antioxidant activity of blackthorn juice with grape juice discovering that the antioxidant of blackthorn juice was higher than the antioxidant activity presented by grape juice (55.13 mg/g DW, respectively 1.15 mg/g DW) [89]. After evaluation of the Folin–Ciocalteu method, FRAP, and ABTS assays, blackthorn extracts showed significant high correlations between antioxidant activities and total phenolic compounds, supporting the theory that polyphenols are the primary contributor to the antioxidant activity of blackthorn fruits [30].

Opriș et al. (2021) studied the antioxidant capacity of blackthorn fruits to integrate the concentrated extracts into the sunscreen formula. The highest content of polyphenols was extracted with ethanol at 40%. Ethanol extract presented antioxidant and UV-protective activities. Blackthorn fruit extracts were applied to two different samples from western and central Serbia. The first sample had a considerably greater total phenolic component content as well as stronger antioxidant activity in DPPH, ABTS, and FRAP assays. However, there were no significant differences between the extracts, implying that, in addition to anthocyanins, other compounds contribute to lipid peroxidation inhibition [35,50]. A recent study on blackthorn leaf extract showed that acetone extract was the most effective in scavenging DPPH and ABTS free radicals (44.57 and 16.12 µg mL^−1^), with acetone revealing the best antioxidant activity. Furthermore, acetone leaf extract was richer in phenols, flavonoids, and anthocyanins than water and ethanol, proving that phenol content and antioxidant activity are closely related [71].

In recent times, another investigation has been conducted to examine the impact of preservation techniques and storage conditions on the antioxidative efficacy of blackthorn phytochemicals. The freeze-drying method showed a significant contribution to increasing the concentration of colored compounds. Although, in terms of antioxidant compound content and bioactivity, lyophilization provided the most favorable storage parameters. Further analysis revealed that the ability of phenolics to reduce iron ions in frozen samples increased during storage [90].

In a study by Ruiz-Rodríguez, De Ancos et al. (2014), the bioactive substances of blackthorn and hawthorn fruits, including vitamin C (ascorbic and dehydroascorbic acids), were evaluated for their antioxidant potential. The results indicated that blackthorn fruits had a total vitamin C content of 11.27 mg/100 g^−1^ fw, with dehydroascorbic acid being the primary contributor. In contrast, hawthorn fruits had a higher total vitamin C content of 30.35 mg/100 g^−1^ fw. Blackthorn fruits also exhibited a higher antioxidant capacity than hawthorn fruits, with the DPPH values indicating a strong correlation with vitamin C and phenolic components. Vitamin C levels in blackthorn fruits were found to be consistent with other study findings, with 20.86 mg/100 g in frozen and 23.84 mg/100 g in fresh raw material. Furthermore, freezing and frozen storage did not significantly alter the antioxidant content of blackthorn fruits [3].

### 4.2. Anti-Microbial and Antifungal Activities

Consumer demand for healthy food, free of synthetic additives has grown recently, generating interest in using natural anti-microbial agents. Blackthorn is used as a medicine by people in countries like Turkey and Serbia to treat bronchial asthma and nephritis [89]. Gündüz (2013) studied the anti-microbial activity of blackthorn purees (fresh and processed) on *Salmonella* spp., this Gram-negative bacteria being reduced about 3.94 and 2.37 log units in fresh and processed purees at 25 °C for 30 min [26]. In accordance, Smullen et al. (2007) demonstrated that extracts containing polyphenols were effective in inhibiting *Streptococcus* mutants, the blackthorn skin extract showing a minimum inhibitory concentration (MIC) of 2 mg/mL [91].

For a more detailed antibacterial and antifungal activity, Veličković I. et al. (2020) tested the different blackthorn extracts on various microorganisms such as *Bacillus cereus*, *Stapylococcus aureus*, *Listeria monocytogenes*, *Escherichia coli*, *Aspergillus niger*, *Penicillium funiculosum*. According to the findings, the ethanol extract was more effective against the tested bacteria, whereas the aqueous extract demonstrated superior antifungal properties [83]. In 2021, the same research group analyzed the blackthorn leaf extract, doing similar tests for antifungal and antibacterial activities using the microdilution method. The pathogenic bacteria examined responded better to the ethanolic extract, especially *E. cloacae* and *B. cereus* [26,71,91]. In contrast to antibacterial activity, the aqueous sample’s antimycotic characteristics were marginally better but still inferior to the positive control (ketoconazole). In both cases, the ethanol and aqueous extracts were chosen due to their non-toxicity. Also, the ethanol extract was richer in flavonoids, which may explain the efficient anti-microbial activity. Furthermore, ethanolic fruit extract obtained by Velickovic et al. (2014) showed anti-microbial activity, using the disc diffusion method, against all tested microorganisms (*S. aureus, E. coli, Salmonella abony,* and *Pseudomonas aeruginosa*) except *Bacillus cereus* [10]. Kumarasamy et al. (2004) confirmed the effectiveness of methanol extracts of blackthorn seeds against *Lactobacillus plantarum, Staphylococcus aureus,* and *Citrobacter freundii*, by using a 96-well microplate-based broth dilution assay [11]. The best result among aerial parts, studied by Dedić et al. (2021), was the leaf extract obtained using MAE, with an inhibition zone of 21.67 mm against *Bacillus subtilis.* However, the fruits from the Trnovo region showed the best results against all the bacterial strains. The aerial parts extracts had the most effective anti-microbial activity against *B. subtilis, S. aureus, E. faecalis,* and *P. aeruginosa* (agar well diffusion method), as well as antifungal activity. The positive controls were streptomycin (10 µg, Oxoid) and respectively antimycotic nystatin (10 µg, Oxoid) [2].

### 4.3. Antidiabetic Effect

Millions of people worldwide suffer from a chronic metabolic condition known as diabetes [22,92]. Based on natural substances, researchers are working to develop a new medication [22]. Because of their variety of benefits, safety, tolerance, and affordability, medicinal herbs like blackthorn may function as an alternative to conventional antidiabetic medications [93]. Genotypes of North Serbian blackthorn, rich in polyphenols with biological activities, were associated with antidiabetic and anti-proliferative effects and antioxidant properties [22]. Also, Stanković et al. (2022) examined the possible anti-diabetic activities of blackthorn ethanolic extracts by testing their ability to inhibit α-glucosidase. As a result, blackthorn extract showed a significant glucosidase inhibitory activity (129.46 ± 0.73). Furthermore, compared to standard anti-diabetic drugs (acarbose), blackthorn extract had a higher IC50 value [34,83].

Moreover, Crnić et al. (2021) examined how blackthorn flower extract affected the glycaemic balance in C57BL/6 mice that were neither normoglycemic nor alloxan-induced hyperglycemic. The data confirmed that short-term (in minutes) or long-term (for 10 days) oral consumption of blackthorn flower extract can slightly but significantly raise blood sugar concentration in metabolically healthy (normoglycemic) mice. Consuming blackthorn flower extract for 10 days reduced blood glucose levels, in C57BL/6 mice, it improved glucose tolerance, boosted insulin production, and reduced serum α-amylase activity, which may have hyperglycemic protective benefits [94].

### 4.4. Anti-Inflammatory Effect

In past studies, different scientists [4,9,10,89,95,96,97] have demonstrated a significant antioxidant activity of blackthorn extracts by 2,2-diphenyl-1-picrylhydrazyl test, knowing the fact that fruits with a high capacity of radical scavenging are associated with low occurrences of degenerative diseases (inflammation and arthritis) [98]. Blackthorn fruit ethanolic extract was investigated for its wound-healing capacity by Coppari et al. (2021) [99]. The human umbilical vein endothelial cells were mechanically scratched within T25 tissue culture flasks and then subjected to evaluation using a phase contrast microscope. The anti-inflammatory features of fruit extract improved wound healing closure by 70%. Magiera et al. (2022) also studied the anti-inflammatory capacity of blackthorn extracts. The fruits from Poland were high in polyphenols, anthocyanins, and flavonols, compounds that contribute to antioxidant and anti-inflammatory capacity. The hydroalcoholic extract showed biological effects such as the secretion of key anti-inflammatory factors (IL-10). These findings may help explain the historic use of blackthorn fruits in treating gastrointestinal chronic inflammatory illnesses, given that natural polyphenols accumulate to a significant degree in the intestines [23].

### 4.5. Anticancer/Antitumoral Effects

A valuable source of phenolic compounds is blackthorn flower, which contains kaempferol, quercetin, kaempferol 3-*O*-l-arabinofuranoside, quercetin 3-*O*-l-arabinofuranoside, kaempferol 3-*O*-l-ramnopyranoside, kaempferol 7-*O*-l-ramnopyranoside, kaempferol 3-*O*-d-x [7,73]. These polyphenols have strong anti-oxidant and pro-oxidant qualities, and they may be used to treat cancer either as a preventative measure (antioxidants) or as a cancer cell killer (prooxidants) [100]. Red grape, blackberry, black cherry, black currant, elderberry, blackthorn, and plum fruit peel polyphenolic extracts led to caspase-dependent cell death in breast cancer MCF-7 cells, which was connected to an increase in oxidative stress and resulted in the release of pro- and anti-apoptotic mitochondrial proteins from the Bcl-2 family [101].

Although Kello et al.’s (2017) focused on the efficiency of blackthorn extracts on breast cancer cells, Murati et al. (2019) exposed human liver cancer cells (HepG2) to various doses of blackthorn flower extract (10–200 g/mL) and measured cytotoxic activity using the neutral red and kenacid blue techniques after 24, 48, and 72 h of incubation to determine its anticancer properties. Significant inhibition of Hep G2 cellular proliferation was observed at concentrations exceeding 50 g/mL, indicating that the blackthorn flower extract may have detrimental effects on human liver cancer cells. The extract’s impact was characterized by apoptosis and necrosis, which were most likely a result of heightened oxidative stress levels [101,102].

According to Condello et al. (2019), the extract of blackthorn fruits combined with a nutraceutical activator complex is well tolerated by mice, inhibits the growth of colorectal cancer, and should be considered for incorporation into multi-drug protocols for colon cancer treatment [103]. More specifically, their research demonstrated the anticancer efficacy of Trigno M, an extract of blackthorn enriched in phenolic acids, flavonoids, and anthocyanins, in combination with the nutraceutical activator complex (NAC), in 2D, 3D, on the reduction of tumor growth, through an in vivo colorectal cancer testing protocol [103].

### 4.6. Other Studies

Balta et al. (2019) investigated tartrazine toxicity development in albino Wistar rats and the protective action of blackthorn fruits. When mixed with benzoates, the synthetic food pigment tartrazine (also known as Yellow 5 or E102) can cause hyperactivity syndrome in children combined with agitation, confusion, and rhinitis [104,105]. The use of tartrazine in groups I, II, and III of rats, led to lesions in all rodents’ kidneys, spleens, and livers, according to histopathological analysis [105]. Tartrazine produced histological abnormalities that led to substantial liver tissue lesions and changes in blood parameters [106]. In this sense, blackthorn powder showed a good protective effect on blood parameters but not offering any discernible benefits for the organs [105].

In another study, Balta V. et al. (2021) examined the extracts’ individual phenolic components for their bioavailability and absorption. They investigated the effects of phenolics from an ethanol-water extract of blackthorn flowers given orally to C57/BL6 mice for 28 days at doses of 25 mg total phenolics/kg body weight. The gut, liver, and kidney phenolic contents were measured using the UPLC-MS/MS technique after 1, 7, 14, 21, and 28 days of extract administration. After consuming blackthorn flower extract for at least three weeks, the number of phenolics in mice tissues considerably increased. In vivo, the extract promoted anti-oxidative defense pathways in an organ-specific manner and possessed significant bioactive properties. The primary components in the gut were 3-O-feruloylquinic acid, 4-O-p-coumaroylquinic acid, kaempherol pentoside, and quercetin rhamnoside, whereas the main components in the liver and kidneys were quercetin 3-O rutinoside, ferulic acid, and 4-O-p-coumaroylquinic acid [107].

Compared to Balta’s study (2021), Dikic et al. (2022) focused on the functionality and bioavailability of polyphenols in blackthorn flower extract (PSE), studying the benefic effects of the extract on the brain. In this regard, a dose of 25 mg polyphenols/kg body weight, was administrated to experimental animals for 28 days. The brain was found to contain a total of 68.7% polyphenols from blackthorn extract. For 59.1% of the chemicals found in the brain, higher (p0.05) C_max_/AUClast values were observed in the PSE treatment group compared to the control group, showing reasonably good bioaccumulation in the brain. The substances that were most abundant in PSE were not always the ones that were most bioabsorbable in the brain. In contrast to phenolic acids, quercetins, or epigallocatechin-3-gallate, kaempferol were not considerably dispersed. On the 28th day, the chemicals with the highest concentrations were 4-p-coumaroylquinonic acid, (-)-epicatechin, quercetin-3-*O*-rutinoside, quercetin-rhamnoside, kaempherol-3-rutinoside, and quercetin-3-glucoside. Researchers concluded that the compounds they had tested would be good candidates for developing or testing “neuro-nutriceuticals”—polyphenol combinations that are specifically targeted at the brain [108].

## 5. Applications

Since ancient times, people have valued blackthorn for treating some stomach affections and the flu. In recent years, they have become more significant as researchers examine them and look for potential applications in various industries. According to Fraternale et al. (2009), these fruits may be useful to the food industry, particularly in the sector that develops dietary supplements and nutraceuticals with enhanced bioactive capabilities [89]. In a recent study, Marcetic et al. (2022) investigated the potential prebiotic effect of blackthorn fruits, noting that the findings are in agreement with those of Milutinovic et al. (2021), who claimed that plants rich in polyphenols have prebiotic activity with a significant impact on *Saccharomyces boulardii* yeast [50,109]. The fact that anthocyanins can prevent the proliferation of harmful bacteria by downregulating gene expression, altering metabolic enzymes, and affecting respiratory metabolism, was also confirmed in a recent study conducted by Wang et al. (2022) [72].

The research conducted by Najgebauer-Lejko et al. (2021) was to obtain probiotic yogurt with 10% sweetened purees of elderberries, sea buckthorn, and blackthorn and assess their chemical composition, acidity, polyphenol, and anthocyanin content. The antioxidant strength and antiradical power were also evaluated. Their high amount of polyphenols, particularly anthocyanins, elderberry, and blackthorn significantly increased the antioxidant capacity of probiotic yogurts. As a result, both prototypes have great potential in acting as natural colorants, the final products turning in a deep purple and stable color. On the other hand, blackthorn puree might be a particularly excellent source of dietary fiber [56].

Blackthorn extract rich in phenolic acids and anthocyanins was encapsulated by Blagojevic et al. (2022) using halloysite and maltodextrin. In this way, the release of bioactive compounds was achieved in a controlled manner using yogurt as a support food, and their bioavailability was significantly improved. Also, these findings suggest that using blackthorn extract and its halloysite and maltodextrin encapsulates form can be a sustainable direction to sustain the development of new functional foods, dietary supplements, and nutraceuticals [110].

Other recent research has highlighted the importance of blackthorn fruit extract in products with solar protection properties. Scientists attempted to obtain various blackthorn polyphenol-rich extracts using intensive treatments such as sonication and test them in sunscreen composition [35,36]. Because blackthorn extract has demonstrated strong antioxidant properties; its in vitro sun protection factor and photostability in the developed sunscreen formulation were also investigated. They concluded that the optimized blackthorn extract can be effectively incorporated into cosmetic formulations increasing its photoprotective properties, and the optimized product remaining stable at the end of the stability study (120 min). These promising results open up new possibilities for the use of blackthorn extract in cosmetic products

Mandic et al. (2018) investigated the effect of blackthorn fruit (*Prunus spinosa* L.) extract on the quality of Kranjska sausage. The results showed that vacuum-packed sausage’s quality and shelf life were greatly improved when natural casings were treated with an aqueous or ethanol extract of blackthorn fruits. The number of lactic acid bacteria on the exterior surfaces of Kranjska sausage filled in casings that had previously been treated with an aqueous or ethanol extract of blackthorn fruits decreased over 60 days of storage in vacuum packs. They concluded that adding blackthorn fruit extract to the sausage filling, where its impact would be more noticeable, would significantly increase the extract’s antioxidant function [37].

For many years, the industrial production of natural-based colorants has consisted primarily of obtaining colorant-rich extracts through the use of conventional heat-assisted extraction with water as a solvent, followed by several drying steps. Procedures used to extract anthocyanins such as ultrasound, microwave, and supercritical fluid-assisted extraction techniques have caught the attention of industrials and researchers recently. Leichtweis et al.’s (2019) objective was to investigate the composition of blackthorn anthocyanins and promote the commercial value of these wild fruits through the development of an anthocyanin-based coloring extract. Moreover, their study aimed to optimize the extraction of anthocyanin compounds from fruit epicarp using methods such as ultrasound extraction and heat-assisted extraction. Ultrasound extraction was shown to be the most effective method, presenting 18.17 mg/g anthocyanin content. By examining extracts rich in anthocyanins that have potential use as natural colorants in several industrial domains, their study helped value blackthorn’s wild fruits. Overall, a workable environmentally friendly method was developed that might be used for small-scale study for industrial manufacturing of colorants based on blackthorn anthocyanins [27].

Furthermore, blackthorn fruit extract was evaluated as natural purple colorant used in doughnut icing. Initially, icing presented a dark purple tone, but after 24 h, the product showed a decrease in color intensity. Despite their color decrease, the antioxidant and antimicrobial activities presented a significant increase, ranking blackthorn extracts as promising candidates as natural food preservatives and colorants [25].

## 6. Conclusions

Blackthorn may be considered a valuable source of bioactive compounds that can maintain human health by preventing or shortening the convalescence period for many disorders due to their antioxidant, anti-inflammatory, antiproliferative, antiviral, and antibacterial properties. Since it has been shown to have health-promoting qualities, blackthorn is now attracting more attention. The potential positive and toxicological effects on the human body are still of great curiosity, even though blackthorn has been well-examined for its content in biologically active compounds. Nevertheless, it still needs to be clarified how to identify the substances specifically responsible for blackthorn’s antibacterial, antiviral, and anticancer properties.

Additionally, the research must be focused on their use as a viable and sustainable alternative to medications, considering consumers’ preferences for introducing more effective plant-based medications to the market and environmentally friendly manufacturing practices. Likewise, consumer demands for safe food free of chemicals have grown, which would make blackthorn anthocyanin extracts excellent as natural food ingredients, enhancing the color and the stability of the products. Therefore, it is imperative to re-evaluate blackthorn fruits as a significant source of safe and cost-effective antioxidants. This plant has the potential to be an excellent source of functional foods, nutritional supplements, and ingredients that prevent lipid oxidation in fat-rich foods. Moreover, as a future perspective, due to already proven antibacterial and antifungal properties, blackthorn extracts could represent an interesting research topic in the development and optimization of innovative and intelligent packaging solutions.

## Data Availability

Not applicable.
